# Virtual differential phase‐contrast and dark‐field imaging of x‐ray absorption images via deep learning

**DOI:** 10.1002/btm2.10494

**Published:** 2023-01-20

**Authors:** Xin Ge, Pengfei Yang, Zhao Wu, Chen Luo, Peng Jin, Zhili Wang, Shengxiang Wang, Yongsheng Huang, Tianye Niu

**Affiliations:** ^1^ School of Science, Shenzhen Campus of Sun Yat‐sen University Shenzhen Guangdong China; ^2^ Institute of Biomedical Engineering Shenzhen Bay Laboratory Shenzhen Guangdong China; ^3^ College of Biomedical Engineering and Instrument Science, Zhejiang University Hangzhou Zhejiang China; ^4^ National Synchrotron Radiation Laboratory University of Science and Technology of China Hefei Anhui China; ^5^ Department of Optical Engineering School of Physics, Hefei University of Technology Hefei Anhui China; ^6^ Spallation Neutron Source Science Center Dongguan Guangdong China; ^7^ Institute of High Energy Physics, Chinese Academy of Sciences Beijing China; ^8^ Peking University Aerospace School of Clinical Medicine, Aerospace Center Hospital Beijing China

**Keywords:** cross‐modality image transfer, deep learning, multi‐contrast CT

## Abstract

Weak absorption contrast in biological tissues has hindered x‐ray computed tomography from accessing biological structures. Recently, grating‐based imaging has emerged as a promising solution to biological low‐contrast imaging, providing complementary and previously unavailable structural information of the specimen. Although it has been successfully applied to work with conventional x‐ray sources, grating‐based imaging is time‐consuming and requires a sophisticated experimental setup. In this work, we demonstrate that a deep convolutional neural network trained with a generative adversarial network can directly convert x‐ray absorption images into differential phase‐contrast and dark‐field images that are comparable to those obtained at both a synchrotron beamline and a laboratory facility. By smearing back all of the virtual projections, high‐quality tomographic images of biological test specimens deliver the differential phase‐contrast‐ and dark‐field‐like contrast and quantitative information, broadening the horizon of x‐ray image contrast generation.

## 1 INTRODUCTION

Cross‐modal learning refers to all forms of learning that can generate information from one or more other modalities. Inaccessible and supplementary information is provided by different types of microscopic, photographic, and radiologic imaging devices. The success of cross‐modality deep learning has been demonstrated in microscopy for staining contrast images from autofluorescence images,^1^ bright‐field contrast images from a single hologram,^2^ special stains from H&E stain,^3^ and super‐resolved images from diffraction‐limited images,^4^ in photography for aberration‐free images from distorted images,^5^ as well as in radiology for synthetic computed tomography (CT) from magnetic resonance imaging (MRI),^6^, ^7^, ^8^, ^9^ high‐quality CT images from sparse‐view projections,^10^, ^11^, ^12^, ^13^ bidirectional MRI and CT transfer^14^ and positron emission tomography from MRI.^15^ The benefits of cross‐modality deep learning usually include savings of economic cost, manpower and data acquisition time, fast outcome delivery, spatial resolution and contrast enhancement, and low radiation dose.

An example of contrast modalities is the interactions of x‐rays with matter through absorption, refraction, and ultra‐small angle scattering, influenced by the thickness, shape, and composition of microstructures. More specifically, absorption contrast is mainly used for imaging high density structures, while phase contrast is more sensitive to minute electron density differences.^16^ Dark‐field contrast, which is primarily caused by ultra‐small angle scattering, provides density variations from sub‐pixel microstructures.^17^ To complement conventional radiography for detecting structures in soft tissues, several x‐ray phase‐contrast imaging techniques have been developed.^18^, ^19^, ^20^, ^21^, ^22^, ^23^ Being compatible with conventional x‐ray tube sources, grating‐based x‐ray interferometric imaging is one of the most promising methods for clinical use.^24^, ^25^, ^26^, ^27^ Nevertheless, this method often suffers from a sophisticated systematic setup in which large‐area and high‐quality gratings are inserted into the rotating gantry, thus necessitates a high level of mechanical stability and measurement accuracy.^28^ Therefore, it is more difficult to acquire high‐quality phase‐contrast and dark‐field tomographic images than standard absorption tomography.

Recently, deep learning networks have been utilized to improve the image quality in grating‐based interferometric imaging.^29^ Furthermore, pseudo differential phase‐contrast (DPC) images have been generated using dual‐energy x‐ray absorption images.^30^ This cross‐modality image transfer between absorption images and DPC images has been demonstrated to be effective when incorporated with the physical imaging model. Nevertheless, this technique is limited to dealing with two‐dimensional DPC images, and requires a mathematical relationship between phase shift signals and dual‐energy absorption coefficients. The normalized scale in their results implies that these procedures are qualitative rather than quantitative. In this work, we report a new approach using deep learning to directly yield virtual DPC and dark‐field projections and tomographic images of high quality. Specifically, multi‐contrast CT scans are employed to obtain the training data set. Cross‐modal learning is performed on each absorption projection using a modified Pix2Pix generative adversarial network (GAN)^31^ that has been trained to match the DPC and dark‐field projection images of a biological specimen after they are paired with the corresponding absorption images. We, thus, bypass complex and sophisticated experimental setup and procedures using a trained neural network to achieve multi‐contrast imaging. The imaging quality of virtual DPC and dark‐field images is demonstrated by applying the trained neural network to absorption projections. The transferred image is compared to the ground‐truth image either obtained at the synchrotron‐based interferometer or the laboratory‐based interferometer. Finally, we show how our approach can directly and efficiently generate high‐contrast visualizations of tomographic images of biological specimens.

## 2 RESULTS

### 2.1 Cross‐modality image transfer

**FIGURE 1:**
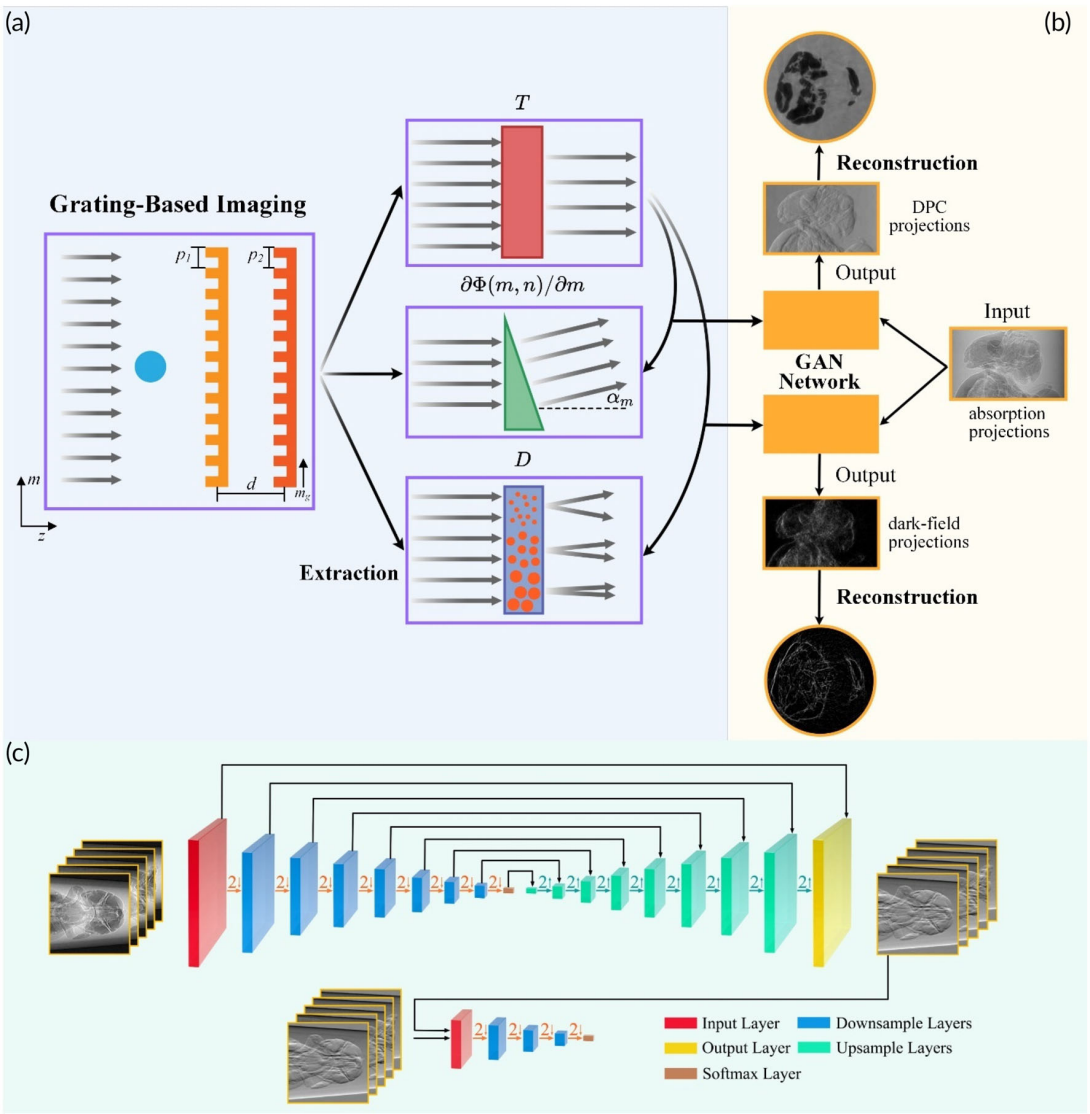
(a) Data acquisition, (b) testing flow, and (c) network training diagram. In contrast to grating‐based imaging, which requires an experimental and physical extraction procedure, a deep neural network can virtually generate differential phase‐contrast and dark‐field images of biological specimens.

The scheme we used to collect the absorption images T, the DPC images $\partial \Phi \left(m,n\right)/\partial m$ and the dark‐field images D, is illustrated in the Figure 1a. A phase‐shift phase grating (G1) and an absorption grating (G2) are placed along the optical axis. The grating lines are oriented parallel to the tomography axis and perpendicular to the plane of the paper. Their separation distance d is defined as the fractional Talbot distance.^32^ According to the classical theory of grating‐based x‐ray interferometric imaging,^17^, ^33^ the interference signal I in the detector coordinates $\left(m,n\right)$ can be expanded in Fourier series:

(1) $$\begin{array}{cc} I\left(m_{g},m,n\right) & =\sum_{i=0}^{\infty }a_{i}\left(m,n\right)\cos\left[2\mathrm{\pi i}\frac{m_{g}}{p_{2}}+\phi_{i}\left(m,n\right)\right]\;\\ & \approx a_{0}\left(m,n\right)+a_{1}\left(m,n\right)\cos\left[2\pi \frac{m_{g}}{p_{2}}+\phi_{1}\left(m,n\right)\right] \end{array},$$

where $m_{g}$ is the lateral shift of G2 relative to G1 along the m direction, $p_{2}$ is the period of G2, $a_{i}$ are the amplitude coefficients and $\phi_{i}$ are the phase coefficients.

In general, the three imaging modalities of absorption T, differential phase $\partial \Phi \left(m,n\right)/\partial m$, and dark‐field D can be retrieved by,

(2) $$T\left(m,n\right)=\frac{a_{0}^{s}\left(m,n\right)}{a_{0}^{r}\left(m,n\right)},$$

(3) $$\partial \Phi \left(m,n\right)/\partial m=\frac{2{\pi \alpha }_{m}}{\lambda }=\frac{p_{2}}{\mathrm{d\lambda}}\left[\phi_{1}^{s}\left(m,n\right)-\phi_{1}^{r}\left(m,n\right)\right],$$

(4) $$D\left(m,n\right)=\frac{V^{s}\left(m,n\right)}{V^{r}\left(m,n\right)}=\frac{a_{1}^{s}\left(m,n\right)}{a_{1}^{r}\left(m,n\right)}\frac{a_{0}^{r}\left(m,n\right)}{a_{0}^{s}\left(m,n\right)},$$

where the subscripts (^*s*^) and (^*r*^) refer to the sample scan and the reference scan, respectively. d is the inter‐grating distance, λ is the wavelength, and $\alpha_{m}$ is the refraction angle with respect to the m‐coordinate. Note that $T\left(m,n\right)$ is the same as what would be measured with a conventional x‐ray radiography setup and is commonly referred to as transmission contrast or attenuation contrast.

The experiments were performed on two grating‐based x‐ray interferometers, one at the BL13W beamline of the Shanghai Synchrotron Radiation Facility (SSRF), China,^34^ and the other at the laboratory at the University of Science and Technology of China (USTC), as described in Section 4.1.

In Figure 1, the deep learning‐based multi‐contrast image generation pipeline is depicted. Through transfer learning from T to $\partial \Phi \left(m,n\right)/\partial m$ and T to D, well‐trained deep neural networks could be built. The neural network architecture of the generator follows the design of U‐net (Figure 1c), and is described in Section 4.3. Once the deep networks have been trained, test data is fed into the networks. Consequently, the neural networks rapidly produce virtual $\partial \Phi \left(m,n\right)/\partial m$ and D projections (the outputs of the networks, the yellow boxes, Figure 1b). Finally, virtual tomographic slices (the outputs of the reconstruction, the yellow circles, Figure 1b) are obtained from virtual projections by using the standard filtered‐back projection algorithm.

### 2.2 Virtual DPC and dark‐field images via a synchrotron interferometer

**FIGURE 2:**
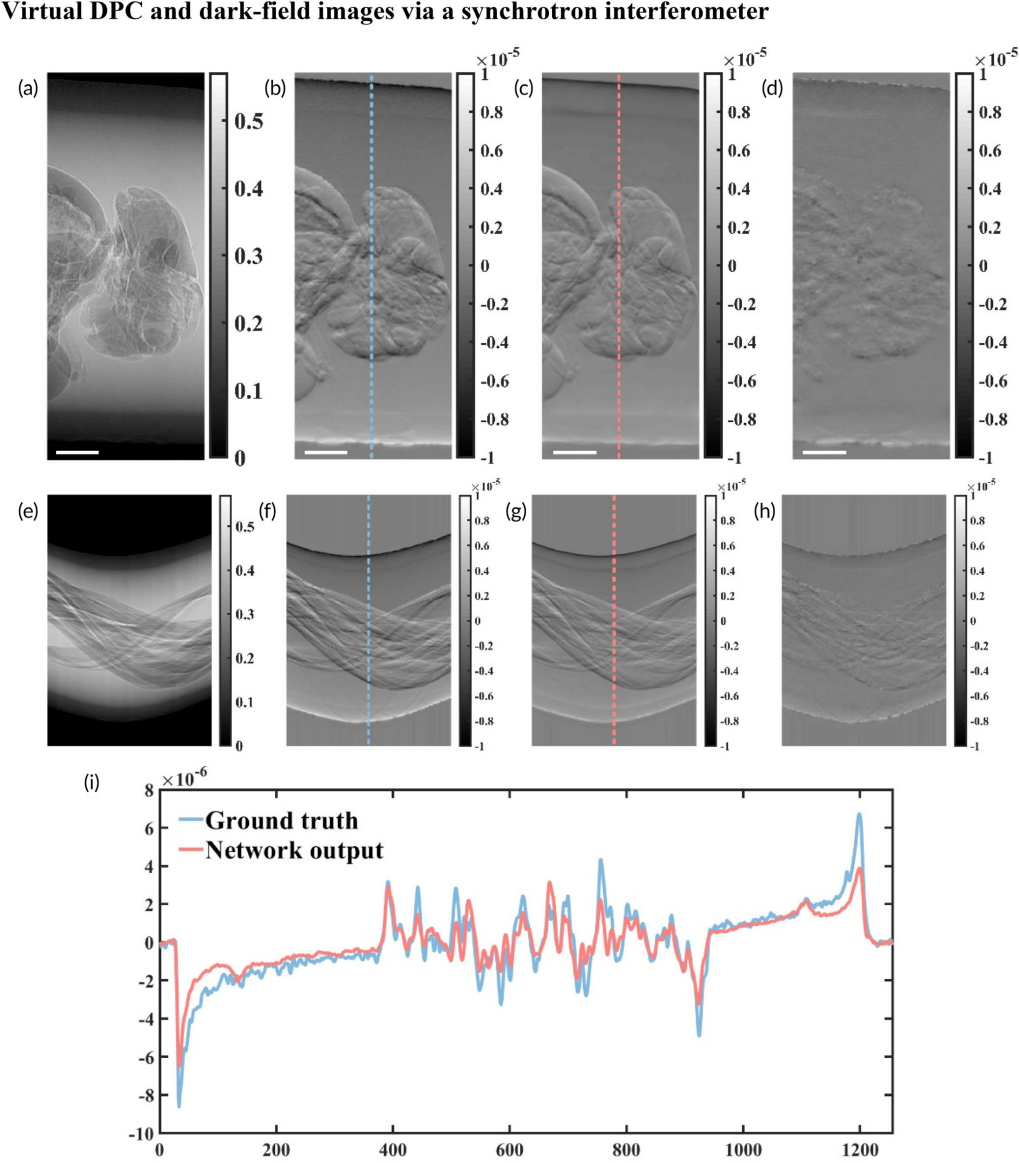
Virtual differential phase‐contrast (DPC) projections match the experimental results. (a–c) Representative projections of a bee sample. (a) An experimental absorption projection used as input into the neural network. (b) An experimental DPC projection acted as the ground truth. (c) A virtual DPC projection (network output) of the same view. (e–g) Sinograms showing the absorption, DPC, and virtual DPC images of the same sample, in order from left to right. (d, h) The error maps (Ground truth − Network output) of the projection and sinogram are provided in 4th column. (i) Selected profiles for comparison. Scale bar, 1 mm (white)

**FIGURE 3:**
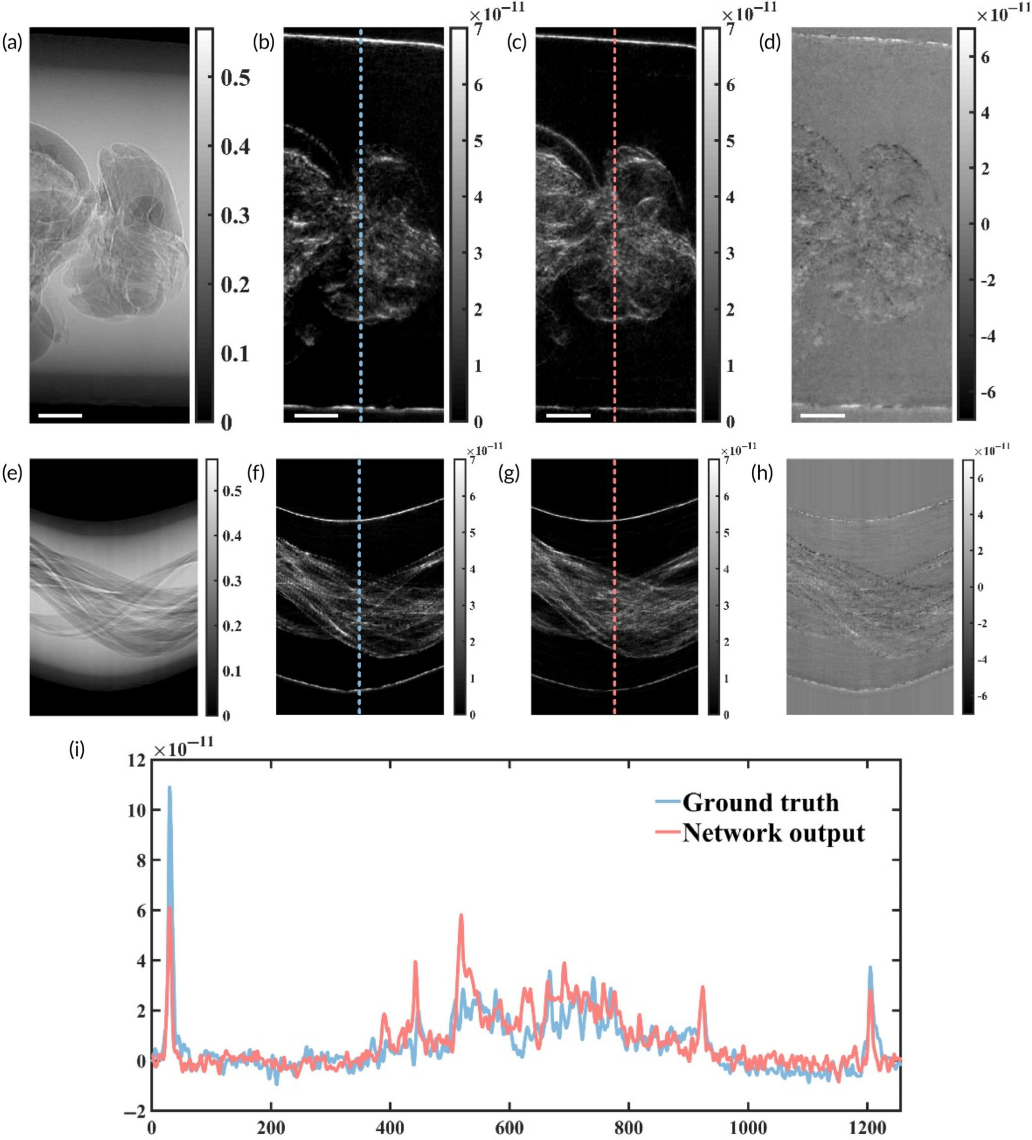
Virtual dark‐field projections match the experimental results. (a–c) Representative projections of a bee sample. (a) An experimental absorption projection used as input into the neural network. (b) An experimental dark‐field projection acted as the ground truth. (c) A virtual dark‐field projection (network output) of the same view. (e–g) Sinograms showing the absorption, scattering, and virtual scattering of the same sample, in order from left to right. (d, h) The error maps (Ground truth − Network output) of the projection and sinogram are provided in 4th column. (i) Selected profiles for comparison. Scale bar, 1 mm (white)

To demonstrate the viability of cross‐modality image transfer, a bee specimen was prepared in our biomedical imaging application. Two bees were fixated in formalin solution over 24 h. Multi‐contrast data sets were acquired using grating‐based imaging in the synchrotron beamline. The training set included 360 paired projections from a CT scan of a bee specimen, bee data set #1. The testing set included 360 paired projections from another CT scan of a fixed bee specimen, bee data set #2. We compared the experimental and virtual projections after training Pix2Pix GAN networks, shown in Figures 2 and 3. Outputs of GAN networks are virtual DPC and dark‐field projections. The representative sinogram of the bee sample results from tomography, in which 360 projections were taken over 180°. In Figure 2, the first row shows the projections (Figure 2a–c) while the second row are sinograms (Figure 2e–g). The DPC profiles of the bee's body are plotted along the dashed lines. In Figure 2i, blue dashed lines in Figure 2b,f are compared to pink dashed lines in Figure 2c,g of the same position. To our surprise, deep‐learning based measurement can achieve differential phase changes on the order of 1 × 10^−7^, that is, −1.027 × 10^−7^ ± 6.23 × 10^−8^ calculated from the DPC error maps (Figure 2d,h), implying that this method could be used as a quantitative reference. These results demonstrate that our proposed method is capable of transforming absorption projections into DPC projections, thereby displaying high‐quality features of soft tissues expected from ground truth.

Soft tissue in biological specimens contributes less to the scattering signal because dark‐field contrast is strongly affected by microscopic density fluctuations. Figure 3 depicts the same view and follows the same layout as Figure 2. The first row of Figure 3 is projections, the absorption projection (Figure 3a), the dark‐field projection (Figure 3b), the virtual dark‐field projection (Figure 3c), and the error map of the ground‐truth with respect to the corresponding virtual dark‐field projection (Figure 3d). The second row of Figure 3 is sinograms (Figure 3e–g), and the error map of the ground‐truth with respect to the corresponding virtual dark‐field sinogram (Figure 3h). The comparison profiles are shown in Figure 3i. It is surprising that the deep neural network could readily learn the boundaries of the bee's inner structure and the interfaces of the plastic tube. In addition, for quantitative measurement, our proposed method achieves a scattering signal accuracy of up to 1 × 10^−12^, that is, −2.381 × 10^−12^ ± 9.18 × 10^−13^ calculated from Figure 3d,h.

**FIGURE 4:**
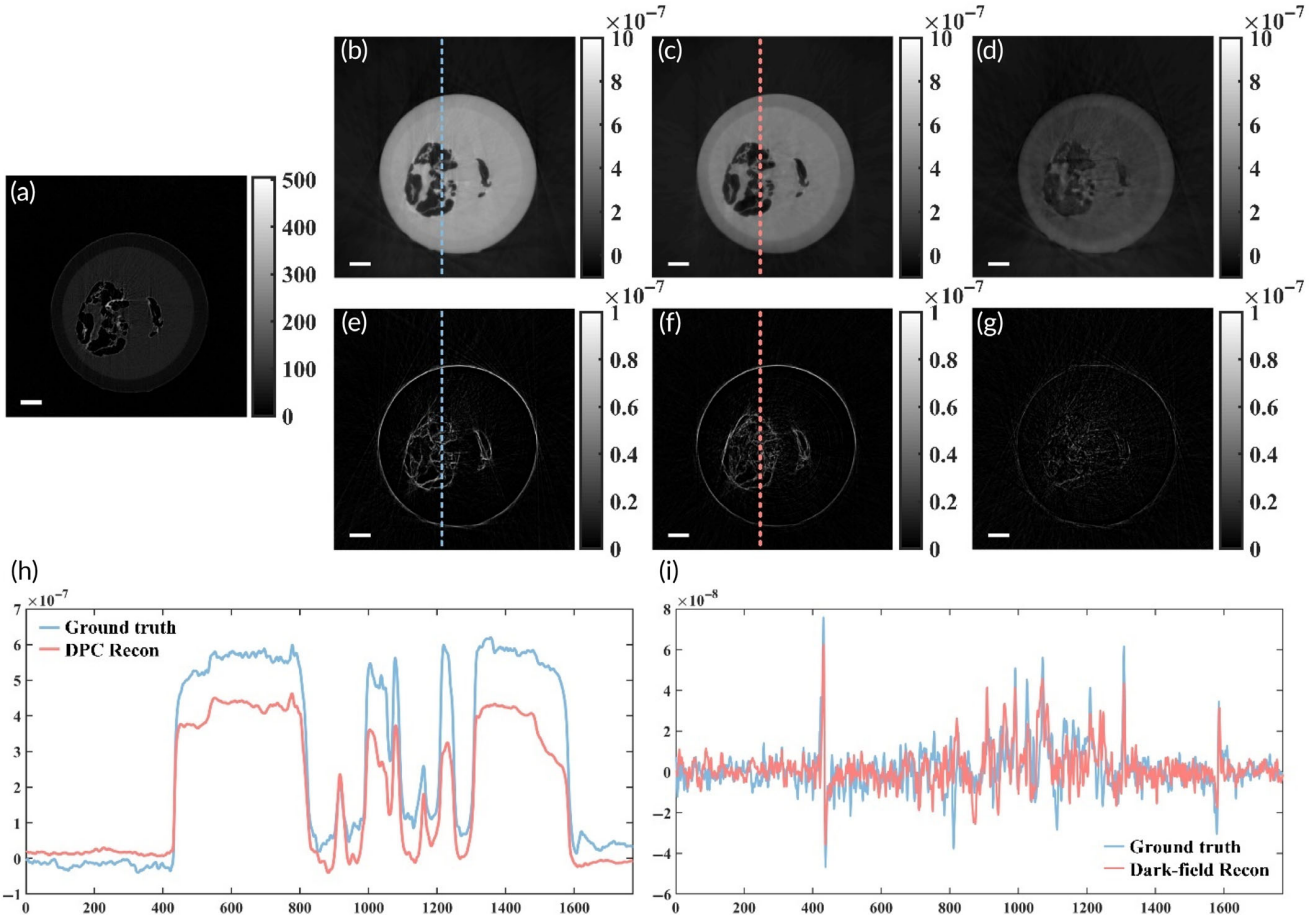
Comparison of virtual differential phase‐contrast (DPC) and dark‐field tomographic slices to ground truths. (a) Representative absorption tomographic slice of a bee sample. (b) An experimental DPC tomographic slice acted as ground truth. (c) A virtual DPC tomographic slice reconstructed from virtual projections. (e) An experimental dark‐field tomographic slice acted as ground truth. (f) A virtual dark‐field tomographic slice reconstructed from virtual projections. (d, g) The error maps (Ground truth − Network output) of the DPC and dark‐field tomographic slice are provided in 4th column. (h, i) Line plots of the ground truth and network output shown in the blue and pink color, respectively. Scale bar, 1 mm (white)

The learning accuracy of the projections could be assessed by the reconstruction quality of the CT system. Improved projections will result in improved reconstructions. For a quantitative comparison, representative tomographic slices of the ground truth, the network output, and the error maps are shown in Figure 4b–g. The absorption slices for the DPC and dark‐field slices are the same (Figure 4a). The virtual DPC slice has a lower amplitude than the experimental slice, as shown in Figure 4h. The effective dark‐field signal is preserved in the virtual tomogram, as shown in Figure 4i. The tomographic slices of the testing data set demonstrate the potential for high‐quality three‐dimensional virtual multi‐contrast imaging.

To further evaluate the generalization ability of cross‐modality image transfer, we used the network that only trains on the bee specimen to test 360 paired projections from a CT scan of a fixated house fly specimen. Figures S1–S4 illustrate the CT results of deep‐learning‐based virtual generation of the house fly. The network outputs match well with the experimental images of the same samples obtained following phase retrieval and dark‐field extraction. These results demonstrate that the deep network can be used to generate both DPC and dark‐field images for different orders of insecta using absorption images. The virtual DPC images capture the local phase gradient of the object. Similarly, the virtual dark‐field images shown in Figure S4 reveal inner boundaries and interfaces between the fly body and the medium, where dark‐field signals were plotted in Figure S4d. The virtual tomographic slices in Figures 2, 3, and Figures S1–S4 accurately depict the multi‐contrast features observed in the experimental images.

To further quantify this comparison, we calculated the peak signal‐to‐noise ratio (PSNR) and structural similarity index (SSIM) values for each network output image with respect to the corresponding ground truth image (Table 1). The DPC projection of Bee #2 has the highest metrics values in most cases, suggesting that the learning accuracy of multi‐contrast images is dependent on the training set and the task at hand. For example, in the case of dark‐field imaging, the fly tomogram is slightly distorted. This is likely due to the fact that the dark‐field image relied on the local scattering power of the sample, which is hard to determine from the absorption image. Figure S1 shows obvious overfitting as a result of the limited generalization ability in the training network. It is worth noting that both the PSNR and SSIM metrics can be affected by background noise in different contrast images. The signal‐to‐noise ratio of experimental dark‐field images is lower than that of experimental DPC images. As a result, the PSNR and SSIM values for the dark‐field image are lower than those for the DPC image.

**TABLE 1:** Comparison of PSNR and SSIM values between the network output images and the corresponding ground truth images

Image type	PSNR		SSIM	
	Average	Standard deviation	Average	Standard deviation
Projection				
DPC (Bee #2)	27.9144	3.3320	0.9511	0.0045
Dark‐field (Bee #2)	24.2259	1.5658	0.7509	0.0253
DPC (Fly)	25.3425	1.7847	0.8793	0.0096
Dark‐field (Fly)	22.7013	1.6145	0.6391	0.0341
Tomogram				
DPC (Bee #2)	22.4350	3.4143	0.8052	0.1751
Dark‐field (Bee #2)	22.2326	0.9839	0.5957	0.0640
DPC (Fly)	18.0814	1.3284	0.5977	0.1693
Dark‐field (Fly)	16.9557	1.9768	0.4275	0.1173

Abbreviations: PSNR, peak signal‐to‐noise ratio; SSIM, structural similarity index.

### 2.3 Virtual DPC and dark‐field images via a laboratory interferometer

**FIGURE 5:**
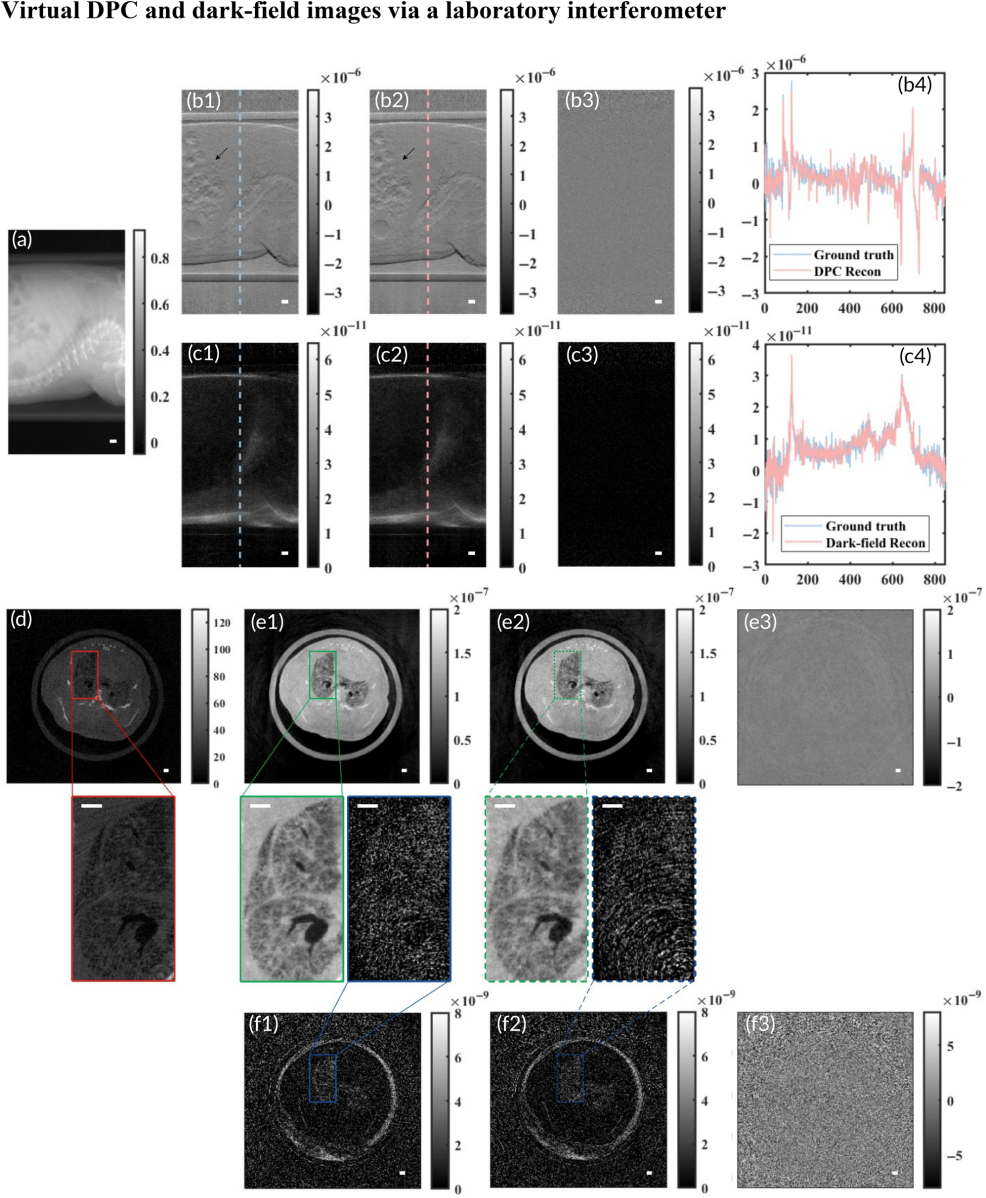
Experimental and virtual‐contrast x‐ray images of a mouse. (a) Representative absorption projection used as input into the neural network and (d) tomographic slice reconstructed by sinogram of absorption projection. (b1–b4) Experimental DPC projection, virtual DPC projection, error map (figure b1 − figure b2), and selected profiles for comparison. (c1–c4) Experimental dark‐field projection, virtual dark‐field projection, error map (figure c1 − figure c2), and selected profiles for comparison. (e1–e3) Experimental DPC tomographic slice, virtual DPC tomographic slice, and error map (figure e1 − figure e2). (f1–f3) Experimental dark‐field tomographic slice, virtual dark‐field tomographic slice, and error map (figure f1 − figure f2). The row between (e) and (f) is close‐up view at 4× magnification on the regions marked in (d, e1, f1, e2, f2). Scale bar, 1 mm (white)

Next, we trained our deep neural network to generate multi‐contrast images virtually using a laboratory x‐ray source. The training set included 190 paired projections from CT short‐scan of three fixed mouse specimens, mouse data set #1–#3, while the testing set included 190 paired projections, mouse data set #4. The laboratory x‐ray source study on the mouse has produced comparable results to the aforementioned beamline study. The inverse Talbot‐Lau design of the grating interferometer, with a microarray anode‐structured target source, results in an extended field of view. Figure 5 summarizes our results for the deep‐learning‐based virtual generation of both projections (Figure 5b2,c2) and reconstruction images (Figure 5e2,f2), which match very well with the experimental results (Figure 5b1,c1,e1,f1). These findings demonstrate that the deep neural network can infer virtual DPC and dark‐field projections, from the corresponding absorption projection. Due to the greater x‐ray attenuation of calcium compared to the lighter organic elements, the conventional x‐ray image (Figure 5a,d) displays very good contrast between bones and soft tissue. In contrast, soft tissue details are clearer in the virtual DPC image (Figure 5b2,e2). As shown by the black arrows in Figure 5b1,b2, the interfaces of the alveolar air region in the virtual DPC projection are consistent with the experimental projection. The error maps of the ground‐truth with respect to the corresponding virtual images (Figure 5b3,c3,e3,f3), selected profiles (Figure 5b4,c4), and metrics values in Table 2 quantitatively compare the difference between ground truth images and virtual images.

**TABLE 2:** Comparison of PSNR and SSIM values between the network output images and the corresponding ground truth images

Image type	PSNR		SSIM	
	Average	Standard deviation	Average	Standard deviation
Projection (Mouse #4)				
DPC	30.3000	1.0808	0.9619	0.0028
Dark‐field	30.0400	1.5175	0.9489	0.0131
Tomogram (Mouse #4)				
DPC	28.6971	1.0529	0.8450	0.0639
Dark‐field	26.1078	1.1786	0.7430	0.0825

Abbreviations: PSNR, peak signal‐to‐noise ratio; SSIM, structural similarity index.

## 3 DISCUSSION AND CONCLUSION

Artificial intelligence (AI) has great potential in medicine,^35^ including assisting to evaluate the efficacy and side effects of potential drugs,^36^ predicting end‐product quality and composition from early time point in‐process measurements during therapeutic cell manufacturing,^37^ and developing an automated in vitro diagnostics platform for an effective feedback control.^38^ Even though the animal specimens used in grating interferometers are disease‐free, the CT results (Figures 4, 5, and Figures S2 and S4) demonstrate the feasibility of using AI to learn absorption projections and use virtual projections to reconstruct. Cross‐modality image transfer can yield additional and reasonable results. Our method has the advantage of requiring only a subset of paired multi‐contrast data for training, and larger absorption images can virtually generate much larger DPC and dark‐field sections. However, this is a preliminary proof‐of‐concept study, and the current approach has several limitations. The main issue we have encountered is caused by the generalization ability, which should be improved further through strategies including early training stopping, L2 regularization, ensembles of networks, etc.

In summary, we present an accurate and reliable method for cross‐modality image transfer in the projection domain, with the goal of generating multi‐contrast images in a direct, convenient, and high‐quality manner. For the first time, we generate DPC and dark‐field style images without the introduction of a physical model. Future research involving big data benefits of AI would be helpful to extend our findings. Ex vivo tissue training related to specific diagnostic contexts and in vivo tissue testing for pre‐clinical evaluation, in particular, could be the next focus. In the presence of pathological processes in breast or lung tissues, this approach may provide radiologists with significantly enhanced contrast compared to routine absorption imaging, as is the case with radiology and CT. In addition, other forms of radiation images could be studied using the cross‐modality image learning method, such as neutrons or atomic particles.

## 4 MATERIALS AND METHOD

### 4.1 Data acquisition

In this study, three types of biological specimens were tested: the Italian bee (Apis mellifera ligustica), the house fly (Musca domestica linnaeus), and the C57BL6 mouse. The bee and fly experiments were conducted at BL13W, SSRF, China, and the mouse experiments were conducted in the laboratory, USTC, China. The bee and fly specimens were placed in a micro‐centrifuge tube filled with formalin.

For the wiggler beamline source, BL13W uses a Si(111) double‐crystal monochromator to produce monochromatic x‐rays with a flux density of ~3.4 × 10^10^ photons/s/mm^2^@20 keV and narrow energy band pass (△*E*/*E* < 5 × 10^−3^, where *E* is the photon energy). The period of a π/2 phase‐shifting grating G1 and an absorption grating G2 are 2.396 and 2.4 μm, respectively. The inter‐grating distance d of the Talbot‐Lau interferometer is 46.4 mm. The pixel number of the detector is 2048 × 2048 and its pixel size is 6.5 μm, resulting in a first‐order visibility of around 40.0% and reconstructed voxel size is 6.5 μm. The acquisition time of a multi‐contrast CT is about 40 min.

For the laboratory x‐ray source, the operation parameters of a microarray anode‐structured target source are 65 kV and 3.8 mA. The period of a π phase‐shifting grating G1 and an absorption grating G2 are 5.08 and 16.6 μm, respectively. The distance between source and G1 is 0.12 m. The inter‐grating distance d of the inverse Talbot–Lau interferometer is 0.68 m. The pixel size of the detector is 91.65 μm, resulting in the first‐order visibility of around 32.0% and the reconstructed voxel size is 39.86 μm. The acquisition time of a multi‐contrast CT is about 3.5 h.

### 4.2 Image processing

We extracted $\partial \Phi \left(m,n\right)/\partial m$ and D data using the conventional phase‐stepping method.^32^ It was accomplished by moving G1 or G2 along the transverse direction perpendicular to both the x‐ray beam and the grating line directions using a step motor with a resolution of 1 nm. G2 was moved by uniformly‐spaced *N* = 6 phase‐steps along the transverse direction $m_{g}$ over one period of the grating, and the image was collected at every step. As already stated in Equation (1), a sinusoidal function fitting can be performed using a Fast Fourier Transform algorithm of the intensity curve $I(m_{g},m,n$) to obtain the offset of the first Fourier coefficient $a_{0}$, amplitudes $a_{1}$, and phases $\phi_{1}$. To retrieve these signals, an analytic form^39^, ^40^ is generally used.

(5) $$a_{0}\left(m,n\right)=\mathrm{abs}\left[\sum_{k=1}^{N}I\left(\frac{k}{N}p_{2},m,n\right)\right],$$

(6) $$a_{1}\left(m,n\right)=\frac{2}{N}\mathrm{abs}\left[\sum_{k=1}^{N}I\left(\frac{k}{N}p_{2},m,n\right)\exp\left(-2\mathrm{\pi i}\frac{k}{N}\right)\right],$$

(7) $$\phi_{1}\left(m,n\right)=\arg\left[\sum_{k=1}^{N}I\left(\frac{k}{N}p_{2},m,n\right)\exp\left(-2\mathrm{\pi i}\frac{k}{N}\right)\right],$$

with the step position *k* ranging from 1…*N* and the G2 grating period $p_{2}$. By taking projections around the sample, the paired $T-\partial \Phi \left(m,n\right)/\partial m$ and T−D at each angle could be acquired. After that, both the experimental projections and virtual projections are reconstructed by the filtered back‐projection method.

### 4.3 Neural network design and implementation

**FIGURE 6:**
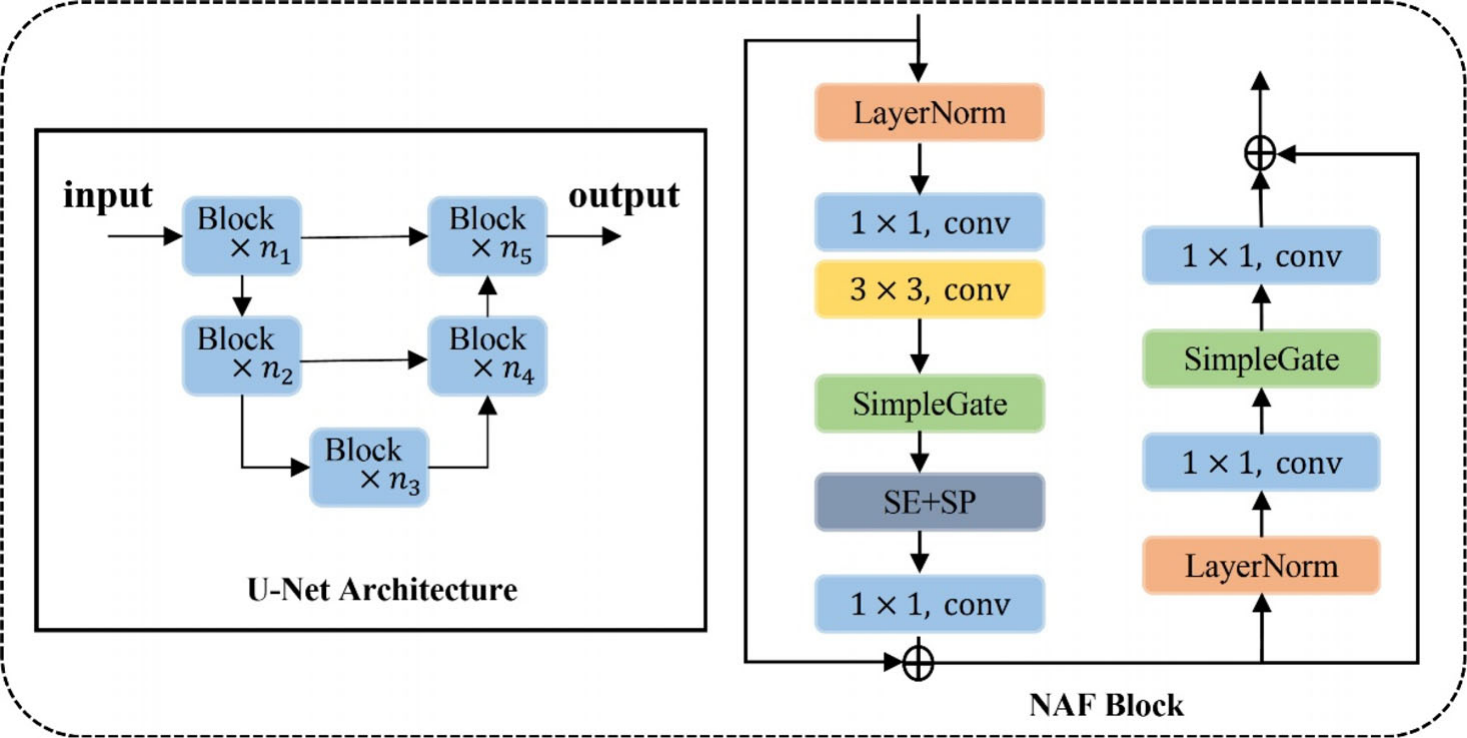
Designed UNet architecture and nonlinear activation free block structure in our network

Figure 1c illustrates a modified version of the Pix2Pix GAN architecture^31^ with eight down‐blocks and eight up‐blocks. The downsample and upsample layers both use a 4 × 4 convolution kernel with a stride of two and one padded zeros, respectively. We replaced the down‐blocks and up‐blocks in Pix2Pix GAN with NAFBlock,^41^ which derives nonlinear activation free (NAF) operations and achieves the state‐of‐the‐art results in image restoration task (Figure 6).

The final objective of our network is:

(8) $$G^{*}=\arg\;\min_{G}\left(\;\max_{D_{1}}\left(L_{\text{LSGAN}}\left(G,D\right)\right)+\lambda L_{L1}\left(G\right)\right),$$

where λ is the coefficient of the regularization term, which is set to 100 in this work to avoid overfitting while ensuring the convergence of the loss function. $L_{\text{LSGAN}}\left(G,D\right)$ represents the loss function of the least‐square GAN. The least absolute error, or L1 norm, was used to regularize the network output. The L1 regularization term is incorporated to constrain the difference between the output of the neural network and the ground truth image. Choosing LSGAN Loss to update has two advantages: first, the outlier fake sample far away from the data set is punished more strictly, which brings the generated image closer to the real data, meanwhile, the image is clearer. The second is the least square, which guarantees greater punishment for the outlier sample, and thereby addresses the problem of insufficient (unstable) conventional GAN training. The learnable variables were updated using the adaptive moment estimation (Adam) algorithm^42^ with a learning rate of 4 × 10^−5^. The training batch size was set at 32.

The network was built using Python 3.6 and Pytorch 1.4.0. Dual NVIDIA GeForce RTX 1080Ti GPUs (Nvidia Corp.) were used for both network training and testing. Data augmentation techniques such as translation and flipping were used to increase the amount of data in the training data set by five times. For example, when 360 image pairs are used for training, the training data set contained 360 × 5 = 1800 image pairs. The training process took around 6 h for 100 epochs.

### 4.4 Evaluation metrics

The PSNR and the SSIM are used to compare the virtual images with ground truth images.^43^ PSNR is an ideal criterion for image quality evaluation, which is defined as:

(9) $$\text{PSNR}=10\log_{10}\left(\frac{{\mathrm{MAX}}_{I}^{2}}{\mathrm{MSE}}\right),$$

where MAX_*I*_ is the maximum value of the ground truth image I. The mean squared error (MSE) between the two images being compared is defined as:

(10) $$\mathrm{MSE}=\frac{1}{M\times N}\sum_{i=0}^{M-1}\;\sum_{j=0}^{N-1}\;{\left[I\left(i,j\right)-I^{\prime }\left(i,j\right)\right]}^{2},$$

where M×N is the pixel number of the image, $I^{\prime }$ is the virtual image compared with the ground truth image.

SSIM is a metric to measure the similarity of two images, which is defined as:

(11) $$\left\{\begin{array}{c} \text{SSIM}\left(a,b\right)=\frac{\left(2\mu_{a}\mu_{b}+h_{1}\right)\left(2\sigma_{a,b}+h_{2}\right)}{\left(\mu_{a}^{2}+\mu_{b}^{2}+h_{1}\right)\left(\sigma_{a}^{2}+\sigma_{b}^{2}+h_{2}\right)}\\ h_{1}={\left(0.01L\right)}^{2}\\ h_{2}={\left(0.03L\right)}^{2} \end{array}\right.,$$

where $\mu_{a}$ and $\mu_{b}$ are the average of *a* and *b* for comparison, $\sigma_{a}$ and $\sigma_{b}$ are the standard deviance of *a* and *b*, $\sigma_{a,b}$ is the covariance of *a* and *b*, and *L* is the dynamic range of the pixel values.

## AUTHOR CONTRIBUTIONS

Xin Ge and Tianye Niu conceived the study. Xin Ge and Pengfei Yang developed the modified network, conducted the training, and testing studies. Xin Ge, Zhao Wu, and Zhili Wang contributed to the imaging studies. Xin Ge and Tianye Niu supervised the overall project and participated in writing the manuscript. The authors read and approved the final manuscript.

## CONFLICT OF INTEREST

The authors declare that they have no competing interests.

### PEER REVIEW

The peer review history for this article is available at https://publons.com/publon/10.1002/btm2.10494.

## Supporting information


**Figure S1.** Representative DPC projections of a fly sample. (a) An experimental absorption projection used as input into the neural network. (b) An experimental DPC projection acted as the ground truth. (c) A virtual DPC projection (output) of the same view. (d) The difference between (c) and (b). (e) Selected profiles for comparison. Scale bar, 1 mm (white).
**Figure S2.** Representative DPC slices of a house fly. (a) A reconstructed transverse slice in experimental absorption tomography. (b) A reconstructed transverse slice in experimental DPC tomography. (c) A reconstructed transverse slice in virtual DPC tomography. (d) Selected profiles for a comparison. Scale bar, 2 mm (white).
**Figure S3.** Representative dark‐field projections of a fly sample. (a) An experimental absorption projection used as input into the neural network. (b) An experimental dark‐field projection acted as the ground truth. (c) A virtual dark‐field projection (output) of the same view. (d) The difference between (c) and (b). (e) Selected profiles for comparison. Scale bar, 1 mm (white).
**Figure S4.** Representative dark‐field slices of a house fly. (a) A reconstructed transverse slice in experimental absorption tomography. (b) A reconstructed transverse slice in experimental dark‐field tomography. (c) A reconstructed transverse slice in virtual dark‐field tomography. (d) Selected profiles for a comparison. Scale bar, 2 mm (white).

## Data Availability

The data that support the findings of this study are available from the corresponding author on reasonable request.
