# Supplementary material for: Virtual differential phase‐contrast and dark‐field imaging of x‐ray absorption images via deep learning
Source: Bioeng Transl Med. 2023 Jan 20;8(6):e10494. doi: 10.1002/btm2.10494 (PMC10658538; doi:10.1002/btm2.10494)
Supplement: Supplementary file 1 — Figure S1. Representative DPC projections of a fly sample. (a) An experimental absorption projection used as input into the neural network. (b) An experimental DPC projection acted as the ground truth. (c) A virtual DPC projection (output) of the same view. (d) The difference between (c) and (b). (e) Selected profiles for comparison. Scale bar, 1 mm (white). Figure S2. Representative DPC slices of a house fly. (a) A reconstructed transverse slice in experimental absorption tomography. (b) A reconstructed transverse slice in experimental DPC tomography. (c) A reconstructed transverse slice in virtual DPC tomography. (d) Selected profiles for a comparison. Scale bar, 2 mm (white). Figure S3. Representative dark‐field projections of a fly sample. (a) An experimental absorption projection used as input into the neural network. (b) An experimental dark‐field projection acted as the ground truth. (c) A virtual dark‐field projection (output) of the same view. (d) The difference between (c) and (b). (e) Selected profiles for comparison. Scale bar, 1 mm (white). Figure S4. Representative dark‐field slices of a house fly. (a) A reconstructed transverse slice in experimental absorption tomography. (b) A reconstructed transverse slice in experimental dark‐field tomography. (c) A reconstructed transverse slice in virtual dark‐field tomography. (d) Selected profiles for a comparison. Scale bar, 2 mm (white). [file BTM2-8-e10494-s001.docx]

**Virtual differential phase-contrast and dark-field imaging of X-ray absorption images via deep learning**

Xin Ge^1,2,+^, Pengfei Yang^3,+^, Zhao Wu^4,+^, Chen Luo^2^, Peng Jin^2^, Zhili Wang^5^, Shengxiang Wang^6,7^, Yongsheng Huang^1^, Tianye Niu^2,8,*^

^1^ School of Science, Shenzhen Campus of Sun Yat-sen University, Shenzhen, China

^2^ Institute of Biomedical Engineering, Shenzhen Bay Laboratory, Shenzhen, Guangdong, China

^3^ College of Biomedical Engineering and Instrument Science, Zhejiang University, Hangzhou, Zhejiang, China

^4^ National Synchrotron Radiation Laboratory, University of Science and Technology of China, Hefei, Anhui, China,

^5^ Department of Optical Engineering, School of Physics, Hefei University of Technology, Anhui, China

^6^ Spallation Neutron Source Science Center, Dongguan, Guangdong, China

^7^ Institute of High Energy Physics, Chinese Academy of Sciences, Beijing, China

^8^ Peking University Aerospace School of Clinical Medicine, Aerospace Center Hospital, Beijing 100049, China

^+^ These authors contribute equally to this work

^*^ [niuty@szbl.ac.cn](mailto:niuty@szbl.ac.cn)


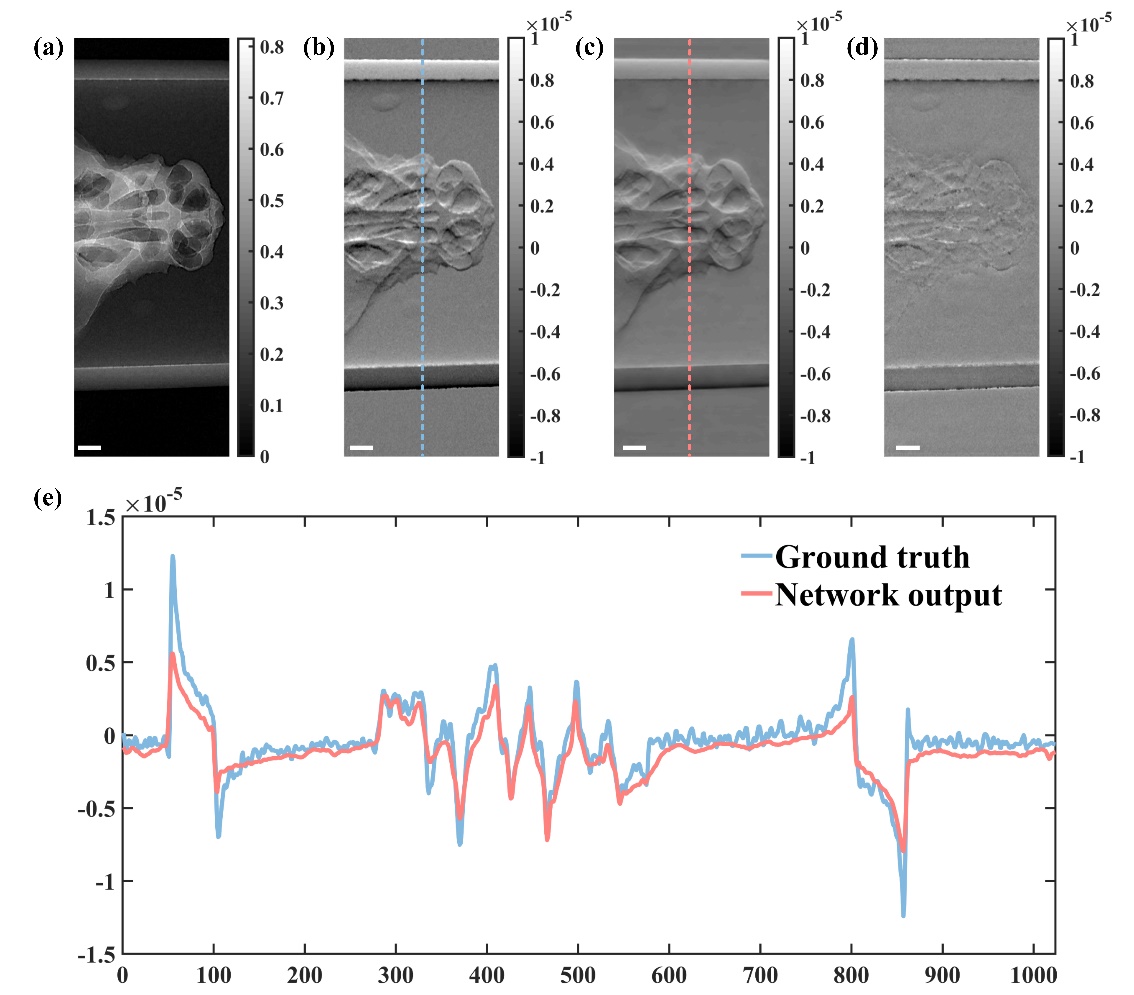


Figure S1 | Representative DPC projections of a fly sample. (a) An experimental absorption projection used as input into the neural network. (b) An experimental DPC projection acted as the ground truth. (c) A virtual DPC projection (output) of the same view. (d) The difference between (c) and (b). (e) Selected profiles for comparison. Scale bar, 1 mm (white).


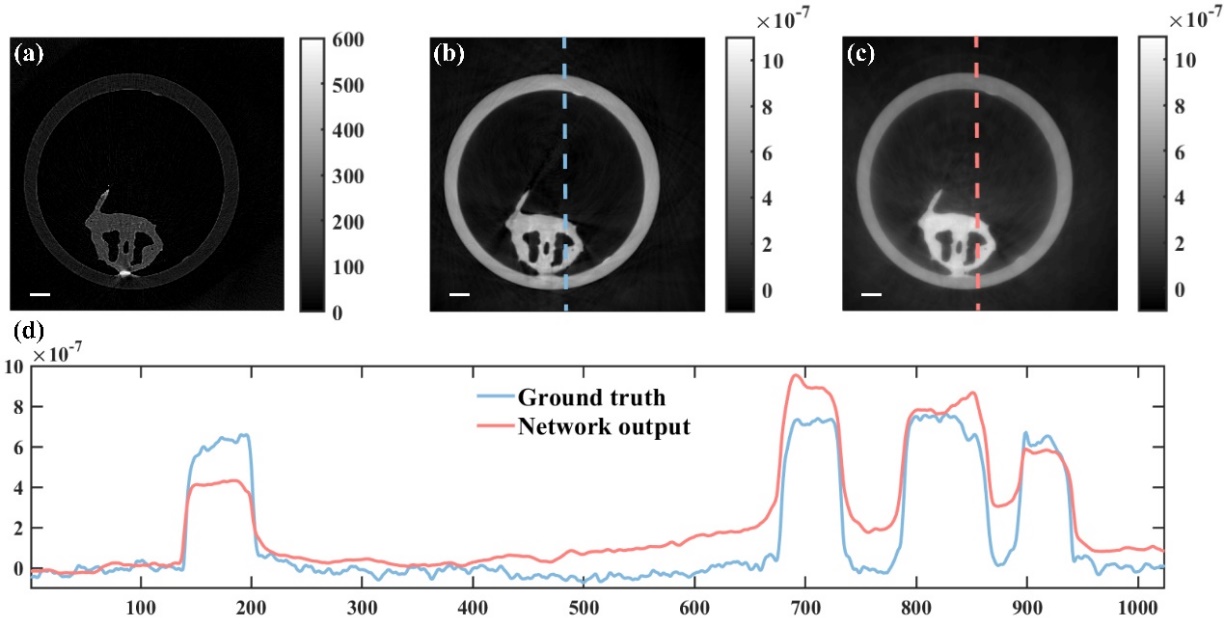


Figure S2 | Representative DPC slices of a house fly. (a) A reconstructed transverse slice in experimental absorption tomography. (b) A reconstructed transverse slice in experimental DPC tomography. (c) A reconstructed transverse slice in virtual DPC tomography. (d) Selected profiles for a comparison. Scale bar, 2 mm (white).


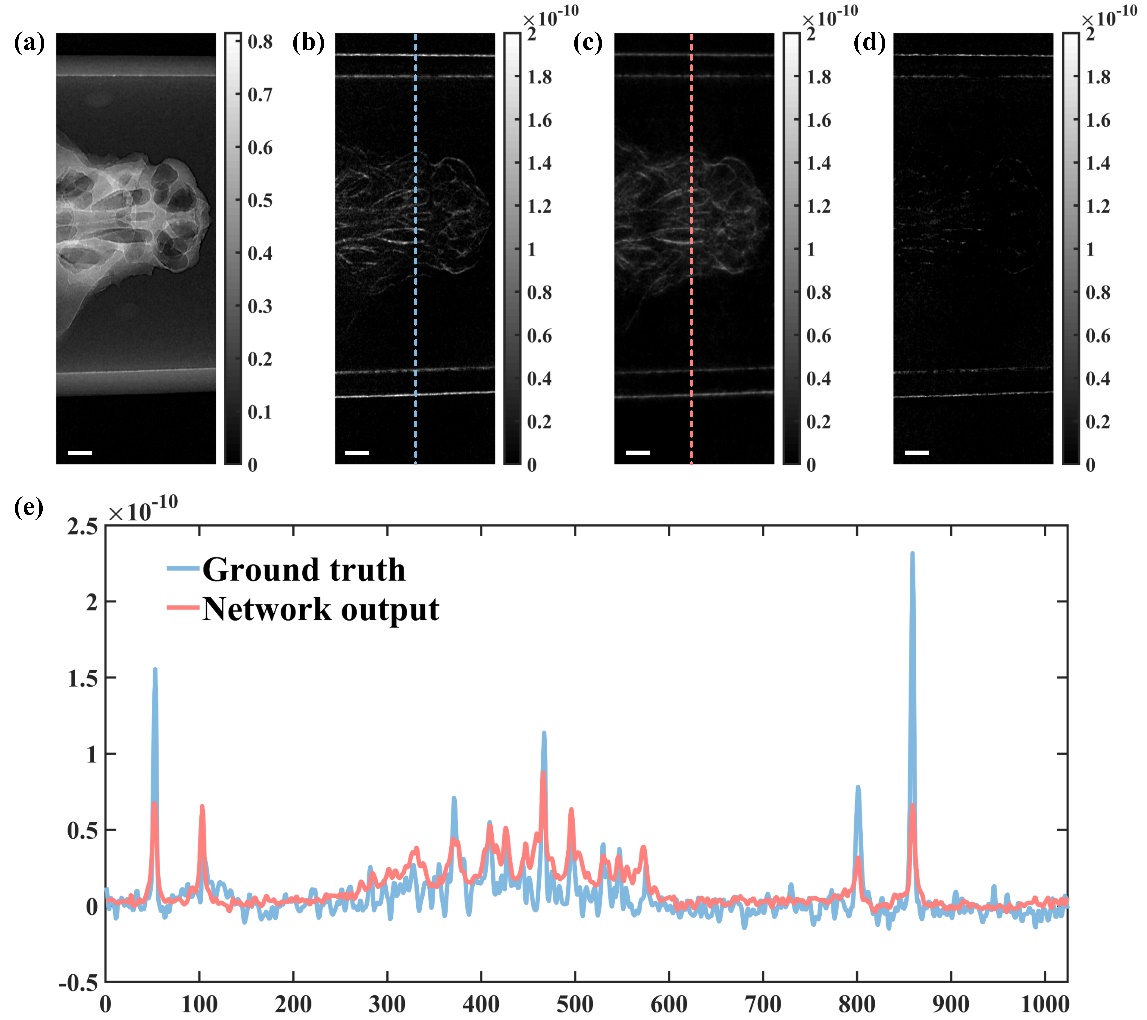


Figure S3 | Representative dark-field projections of a fly sample. (a) An experimental absorption projection used as input into the neural network. (b) An experimental dark-field projection acted as the ground truth. (c) A virtual dark-field projection (output) of the same view. (d) The difference between (c) and (b). (e) Selected profiles for comparison. Scale bar, 1 mm (white).


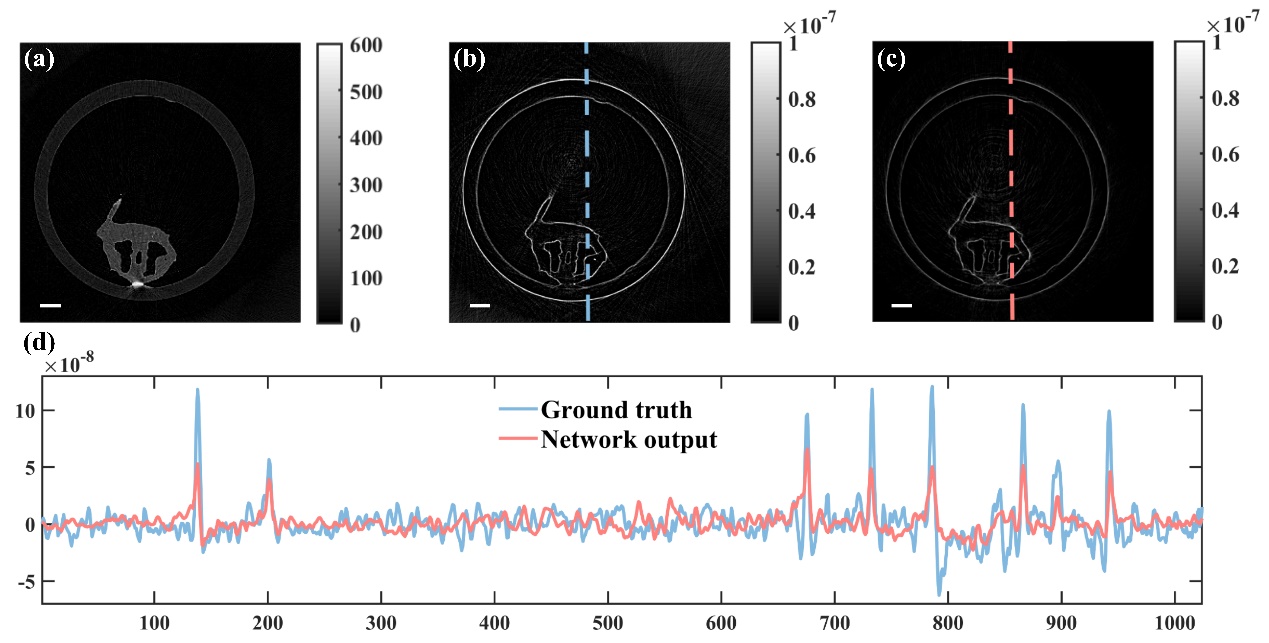


Figure S4 | Representative dark-field slices of a house fly. (a) A reconstructed transverse slice in experimental absorption tomography. (b) A reconstructed transverse slice in experimental dark-field tomography. (c) A reconstructed transverse slice in virtual dark-field tomography. (d) Selected profiles for a comparison. Scale bar, 2 mm (white).
